# Long-term UVA exposure to the eye compromises memory and learning ability in mice via corticotropin-releasing hormone type 2 receptor

**DOI:** 10.7150/ijbs.45967

**Published:** 2020-05-18

**Authors:** Keiichi Hiramoto, Yurika Yamate

**Affiliations:** Department of Pharmaceutical Sciences, Suzuka University of Medical Science, Suzuka, Mie, Japan

**Keywords:** Ultraviolet A, Memory and learning ability, Reactive oxygen species, Corticotropin-releasing hormone, Urocortin 2, Microglia

## Abstract

Long-term eye exposure to ultraviolet (UV)A can effect memory and learning ability. However, the underlying mechanism behind these effects remain unknown. In this study, we used HR-1 mice to study effects of long-term UVA eye irradiation. The eyes or dorsal skin of the mice were exposed to UVA at the dose of 110kj/m^2^ using an FL20SBLB-A lamp three times a week over 12 months. We measured the levels of reactive oxygen species, corticotropin-releasing hormone (CRH), urocortin 2, and CRH type 2 receptor (CRHR-2) in the brain of treated and control animals. Their memory and learning ability following exposure to UVA was analyzed by the standard water maze test. Our results showed that the levels of reactive oxygen species, CRH, urocortin 2, and CRHR-2 increased significantly following long-term UVA irradiation, and the effects were more pronounced in animals subjected to eye irradiation than those subjected to dorsal skin irradiation. Furthermore, the UVA exposure led to an increase in the levels of β-amyloid and microglia in the brain. These results indicated that UVA eye irradiation potentially mediated a decline in memory and learning ability via enhancing levels of urocortin 2, microglia, and β-amyloid in the brain.

## Introduction

Exposure to ultraviolet (UV)A rays is known to exert numerous effects on living beings. Recent scientific research has shown that prolonged UVA exposure induces photoaging and skin cancer. UV irradiation directly activates cell membrane receptors, such as epidermal growth factor receptor, interleukin (IL)-1 receptor, and tumor necrosis factor (TNF)-α receptor. Cells irradiated by UV rays exhibit an increased generation of reactive oxygen species (ROS), such as hydrogen peroxide, superoxide ions, and singlet oxygen, that can lead to DNA damage.

Vale et al. identified corticotropin-releasing hormone (CRH) as a hypothalamic peptide that plays a central role in the body's neuroendocrine response to various types of stress 1. Physiologically, CRH is involved in fear, anxiety, and awakening responses, and also affects the immune and circulatory systems. The actions of CRH are mediated by two types of CRH receptors (CRHR), namely CRHR-1 and CRHR-2, which are G protein coupled receptors (GPCR) 2. Urocortins 1, 2, and 3 are CRH-like peptides and display high affinity to CRHR-2. Together, CRH and urocortins are termed as “stress peptides” and integrate intricate responses of autonomic, endocrine, immune, and cardiovascular systems. The mRNA levels of urocortin increase in the rat paraventricular nucleus in response to stress 3-6. An earlier study published by our group showed that UVA irradiation of the eye is related to CRH and urocortin 2 expressions in the brain 7.

The microglia and β-amyloid peptide in the brain are associated with memory and learning ability. β-Amyloid is a 36-43 amino acid peptide is the main component of amyloid plaques found in the brain of patients with Alzheimer's disease 8. β-Amyloid is derived from the cleavage of amyloid precursor protein (APP) mediated by β- and γ-secretase 9. The microglia, the primary innate immune effector cells of central nervous system (CNS) modulate various functions in central nervous system and regulate the production and release of cytokines and chemokines. Microglia are activated by nerve damage, stress, and intracellular infection, and release inflammatory cytokines such as tumor necrosis factor (TNF)-α, IL-1β, and IL-6 resulting in degeneration and inflammation of the CNS 10. It has been postulated that the microglia-derived inflammatory cytokines may cause functional impairment in the CNS and aggravate pathological conditions such as multiple sclerosis and Alzheimer's disease 11.

Long-term irradiation with UVA has been reported to induce photoaging of the skin 7, while long-term UVA irradiation of the eye can lead to oxidative stress in the brain. The oxidative stress in the brain can, in turn, induce the expression of CRHR, activates hypothalamus-pituitary-adrenal (HPA) axis and activates the central stress response system 7.

Our previous report showed that the long-term UVA irradiation of the eye suppresses memory and learning ability. Long-term exposure to UVA radiation leads to the decrease in the brain acetylcholine levels and a concomitant accumulation of β-amyloid and advanced glycation end products (AGEs) 12. However, the underlying mechanism of the UVA irradiation on reduced memory and learning ability remains unknown. This study was aimed at elucidating the relationship between the long-term UVA eye irradiation and compromised memory and learning ability.

## Materials and Methods

### Animal experiments

This study was carried out by strictly following the recommendations and guidelines for the care and use of laboratory animals at Suzuka University of Medical Science (approval number: 34). All surgical procedures were performed under pentobarbital anesthesia and every effort was made to minimize the suffering of the animals. We used 8-weeks-old, specific-pathogen-free (SPF) hairless HR-1 mice (SLC, Hamamatsu, Shizuoka, Japan) for this study. The mice were individually housed in cages in an air-conditioned room at 23 ± 1°C under SPF conditions with a 12:12 h light-dark cycle. There were 10 mice each in the following groups: control group, UVA dorsal skin irradiated group, and UVA eye irradiated group. UVA irradiation was performed as described previously 13. Briefly, the eyes or dorsal skin were irradiated with UVA (wavelength: 320-400 nm) at the dose of 110 kJ/m^2^ using an FL20SBLB-A lamp (wavelength: 320-400 nm, peak emission: 352 nm; Toshiba Co., Tokyo, Japan) under light Nembutal anesthesia 12. UVA light was passed through a glass filter to block UVB rays. During irradiation, the surface area of the animal, other than the eye and dorsal skin, was covered with aluminum foil. The control group was irradiated similarly using visible light (wavelength: 400-700 nm). The animals were irradiated three times each week over the one year period. No signs of injury to the eye were observed with the doses of UVA irradiation administered.

### CRHR 2 antagonist treatment

Animals were subjected to intracerebral administration of CRHR 2 antagonist antisauvagine-30 (100 mg/kg body weight) (R&D Systems, Minneapolis, MN, USA) in saline, three times per week for one year; the control animals were administered saline 14.

### CRHR 2 agonist treatment

Animals were subjected to intracerebral administration of CRHR 2 agonist urocortin 2 (Peptide Institute, Ibaraki, Osaka, Japan) (100 mg/kg body weight) in saline three times per week for one year; the control animals were administered saline 14.

### N-acetylcysteine (NAC) treatment

Animals were subjected to intracerebral administration of hydroxyl radical scavenger NAC (Nacalai Tesque, Kyoto, Japan) (200 mg/kg body weight) in 0.1% dimethyl sulfoxide (DMSO) three times per week for one year; the control animals were administered 0.1% DMSO 15.

### Open field test

Motor activities of the animals were quantified with an open field apparatus (50 cm (W) x 50 cm (D) x 40 cm (H)) lighted at 70 lux for recording the total distance traveled (cm). Data were analyzed over 15 min period using a video-tracking system (Smart 2 software, Panlab, Barcelona, Spain).

*Behavioral test.* The water maze test was adapted from a previously published method of Morris 16. Briefly, the mice were trained in two slots of four trials on all days of every test week. During the test, each animal remained on the platform for a 60-s interval. To evaluate the memory and learning ability, the swimming time was measured from being thrown in to the water to reaching the platform (goal latency) during each trial.

### Measurement of CRH, urocortin 2, and ROS in the whole brain

Following UVA eye irradiation for 12 months, we measured the CRH, urocortin 2, and ROS levels in the whole brain of the mice. The levels of CRH and urocortin 2 were measured using ELISA assay kits (Yanaihara Co., Ltd., Shizuoka, Japan) according to the manufacturers' instructions. The ROS levels were determined using an OxiSelect^TM^ In Vitro ROS/RNS Assay kit (STA-347; Cell Biolabs, Inc., San Diego, CA, USA) according to be manufacturer's instructions.

### Western blot analysis of the whole brain

The whole-brain samples were homogenized in lysis buffer (Kurabo, Osaka, Japan) and centrifuged at 8000 x g for 10 min. The supernatant from each sample was separated on SDS-PAGE and western blot was performed as described previously 17. Briefly, 10 μg of protein was separated by electrophoresis and transferred to nitrocellulose membranes. Subsequently, the membranes were incubated at 25°C for 1 h with the following primary antibodies; against CRHR-1 (1:1000; GeneTex Inc., Irvine, CA, USA), CRHR-2 (1:1000; Novus Biologicals, Littleton, CO, USA), β-amyloid (1:1000; Rackland Inc., Gilbertsville, PA, USA), Iba (marker of microglia, 1:1000, Wako, Osaka, Japan), IL-6 (1:1000; Abcam, Cambridge, UK), F4/80 (marker of macrophages, 1:1000, Abcam) or β-actin as the loading control (1:5000; Sigma-Aldrich, St. Louis, MO, USA). Next, the membranes were treated with horseradish peroxidase-conjugated secondary antibody (1:1000; Novex, Frederick, MD, USA), and the immune complexes were detected using ImmunoStar Zeta regent (Wako). The images were acquired using the Multi-Gauge software program (Fujifilm, Greenwood, SC, USA).

### Statistical analyses

All data are presented as the means ± standard deviation (SD). The statistical significance of the data was analyzed using Microsoft Excel 2010, with a one-way analysis of variance (ANOVA) followed by Tukey's post hoc test being performed using SPSS version 20 (IBM, Armonk, NY, USA). Differences were considered statistically significant at *p* < 0.05.

## Results

### Behavioral effects of long-term UVA irradiation in mice

**Fig 1:**
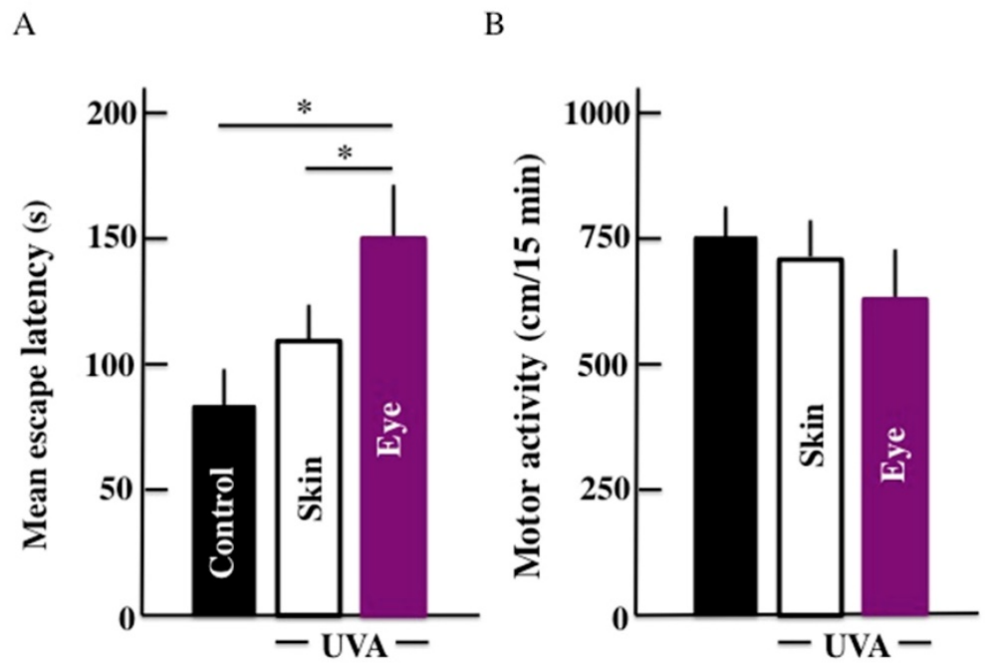
Effects of long-term UVA irradiation on the memory and learning ability (A) and motor activity (B) in the mice. We used the Morris water maze for measuring the memory and learning ability. Values are expressed as the means ± standard deviation (SD) derived from ten animals. *p < 0.05.

The results for the mean escape latency from the Morris water maze are shown in Fig. 1A. Our results showed that the mean escape latency of UVA irradiated groups was higher than the control group. The mean escape latency was significantly higher in mice subjected to UVA eye irradiated mice than those UVA skin irradiated mice. Further, no significant difference in the mean escape latency was observed between UVA skin irradiated mice and the control group. Surprisingly, we did not find any significant difference among the three groups in the results of the motor activity by open field test (Fig. 1B).

### Effects of long-term UVA irradiation on ROS, CRH, and urocortin 2 levels in the whole brain

**Fig 2:**
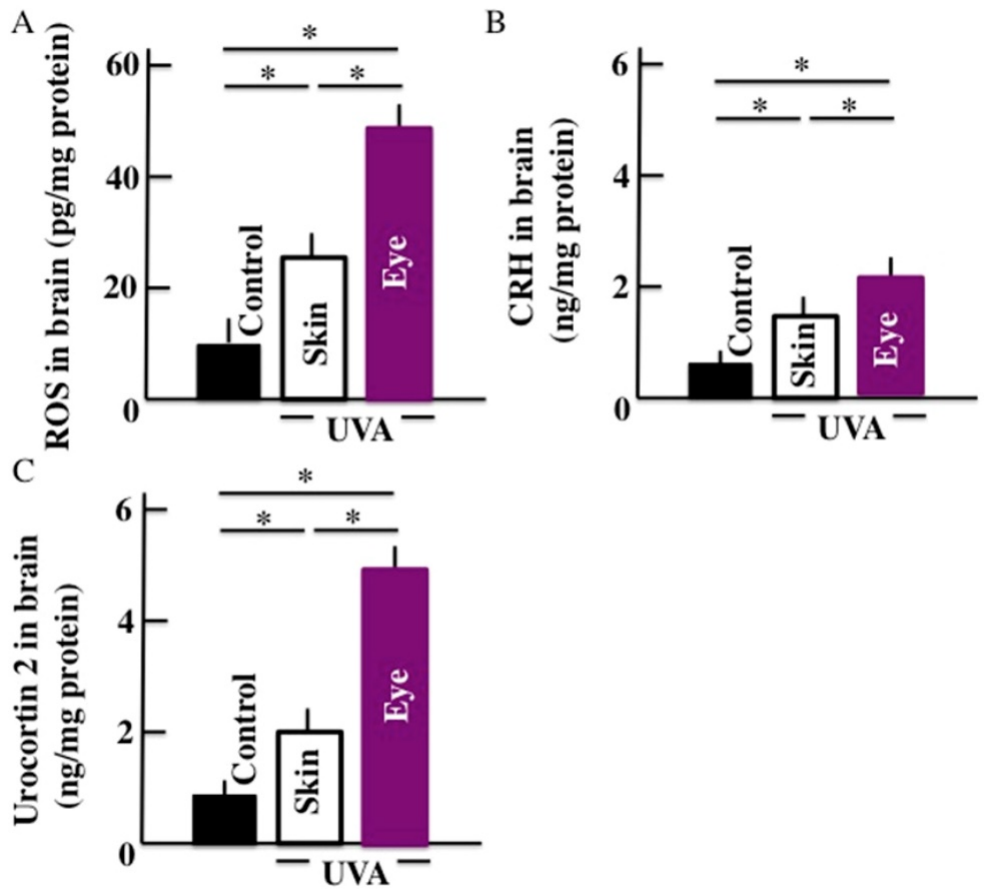
Effects of long-term UVA irradiation on the ROS (A), CRH (B), and urocortin 2 (C) levels in the brain. Values are expressed as the means ± SD derived from ten animals. *p < 0.05.

Our results showed that the levels of ROS (Fig. 2A), CRH (Fig. 2B), and urocortin 2 (Fig. 2C) increased significantly in the brains of animals subjected to the long-term UVA irradiation. Further, animals subjected to UVA eye irradiation presented significantly higher levels of ROS, CRH, and urocortin 2 than animals subjected to UVA skin irradiation.

### Effect of long-term UVA irradiation on CRHR-1, CRHR-2, β-amyloid, and microglia expression in the whole brain

**Fig 3:**
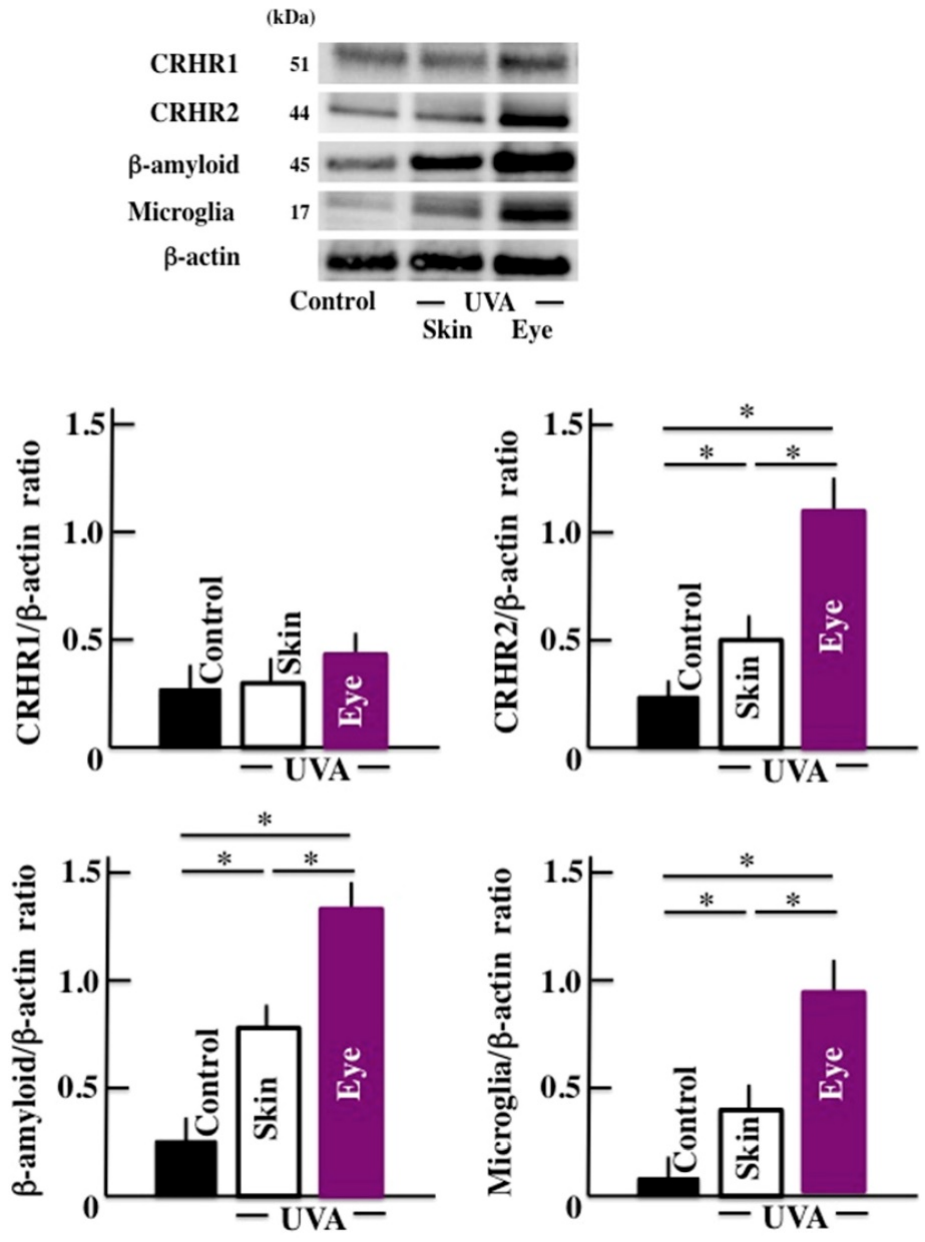
Effects of long-term UVA irradiation on the expression of CRHR-1, CRHR -2, β-amyloid, and microglia in the brain. Values are expressed as the means ± SD derived from ten animals. *p < 0.05.

We observed that the levels of CRHR-2, b-amyloid, and microglia increased significantly in the brain tissue of the animals exposed to long-term UVA irradiation (Fig. 3). Once again, the UVA eye irradiation group presented higher levels of these markers compared to the UVA skin irradiation group. However, no significant difference was observed in the levels of CRHR-1 in any of the treatment groups.

### Effect of long-term UVA irradiation on IL-6 and macrophages in the brain

**Fig 4:**
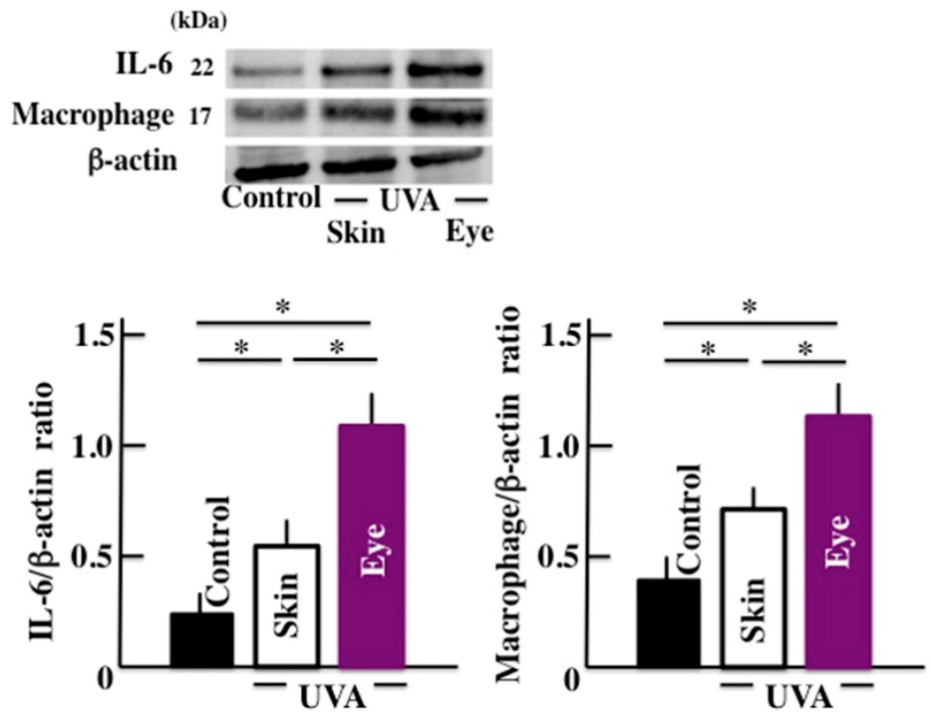
Effects of long-term UVA irradiation on the expression of IL-6 and macrophages in the brain. Values are expressed as the means ± SD derived from ten animals. *p < 0.05.

Next, we examined the levels of IL-6 and the macrophage which are closely concerned in the microglia. The expression of IL-6 and macrophage in brain significantly increased by long-term UVA irradiation (Fig. 4). Here too, we observed a higher response in animals subjected to UVA eye irradiation than those subjected to UVA skin irradiation.

### Effect of CRHR-2 antagonist (Antisauvagine-30) on the mean escape latency and β-amyloid levels in the brain

**Fig 5:**
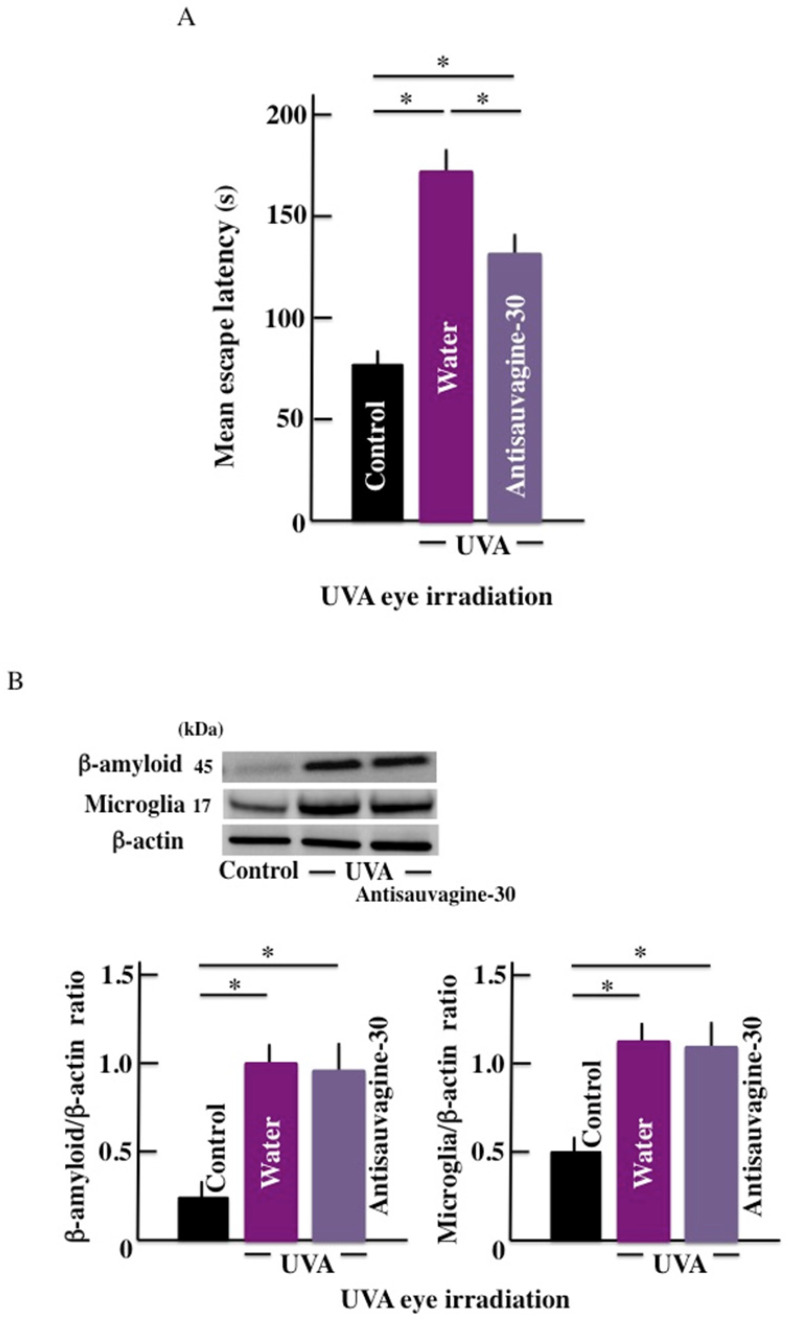
Effects of CRHR-2 antagonist (Antisauvagine-30) treatment on long-term UVA eye irradiated mice. (A) memory and learning ability (mean escape latency), and (B) the expression of β-amyloid and microglia in the brain. Values are expressed as the means ± SD derived from ten animals. *p < 0.05. Scale bar = 100 μm.

The mean escape latency significantly increased by long-term UVA eye irradiation (Fig. 5A). However, treatment with the CRHR-2 antagonist led to a decrease in the mean escape latency when compared with the control group. Further, long-term UVA eye irradiation caused higher expression of the β-amyloid in the brain of the treated mice than the control mice. However, we did not observe any difference in the expression of b-amyloid in the presence or absence of the antagonist treatment (Fig. 5B).

### Effect of CRHR-2 agonist (urocortin 2) on the mean escape latency and brain β-amyloid level

**Fig 6:**
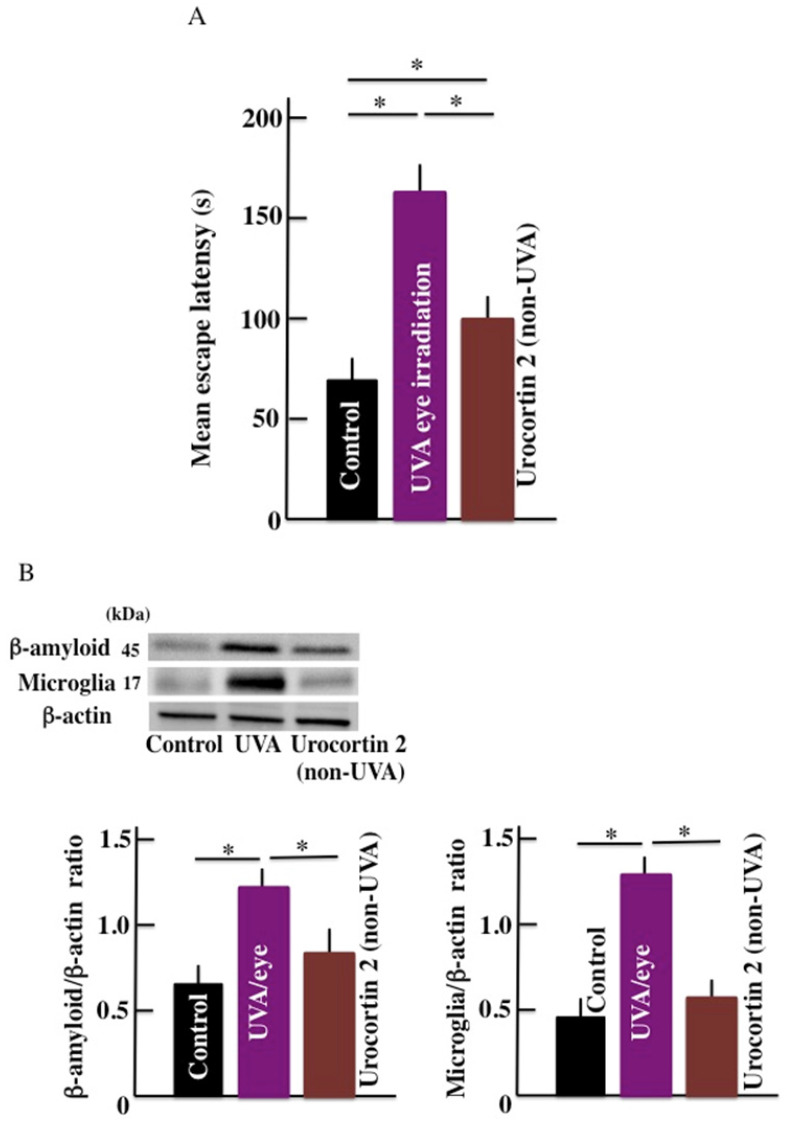
Effect of CRHR-2 agonist (urocortin 2) treatment on long-term UVA eye irradiation mice. (A) memory and learning ability (mean escape latency), and (B) the expression of β-amyloid and microglia in the brain. Values are expressed as the means ± SD derived from ten animals. *p < 0.05.

Compared with the control mice, the mean escape latency was observed to be high in the UVA eye irradiated mice and non-irradiated urocortin 2 administered mice. Furthermore, the mean escape latency of urocortin 2 administered mice was lower than UVA eye irradiated mice (Fig. 6A). The expression of b-amyloid and microglia markers in the brain of UVA eye irradiated mice were significantly higher than the control mice. We did not observe any difference in the expression of the b-amyloid and microglia markers (Fig. 6B) in urocortin 2 injected and control mice.

### Effect of hydroxyl radical scavenger (NAC) administration on the mean escape latency

**Fig 7:**
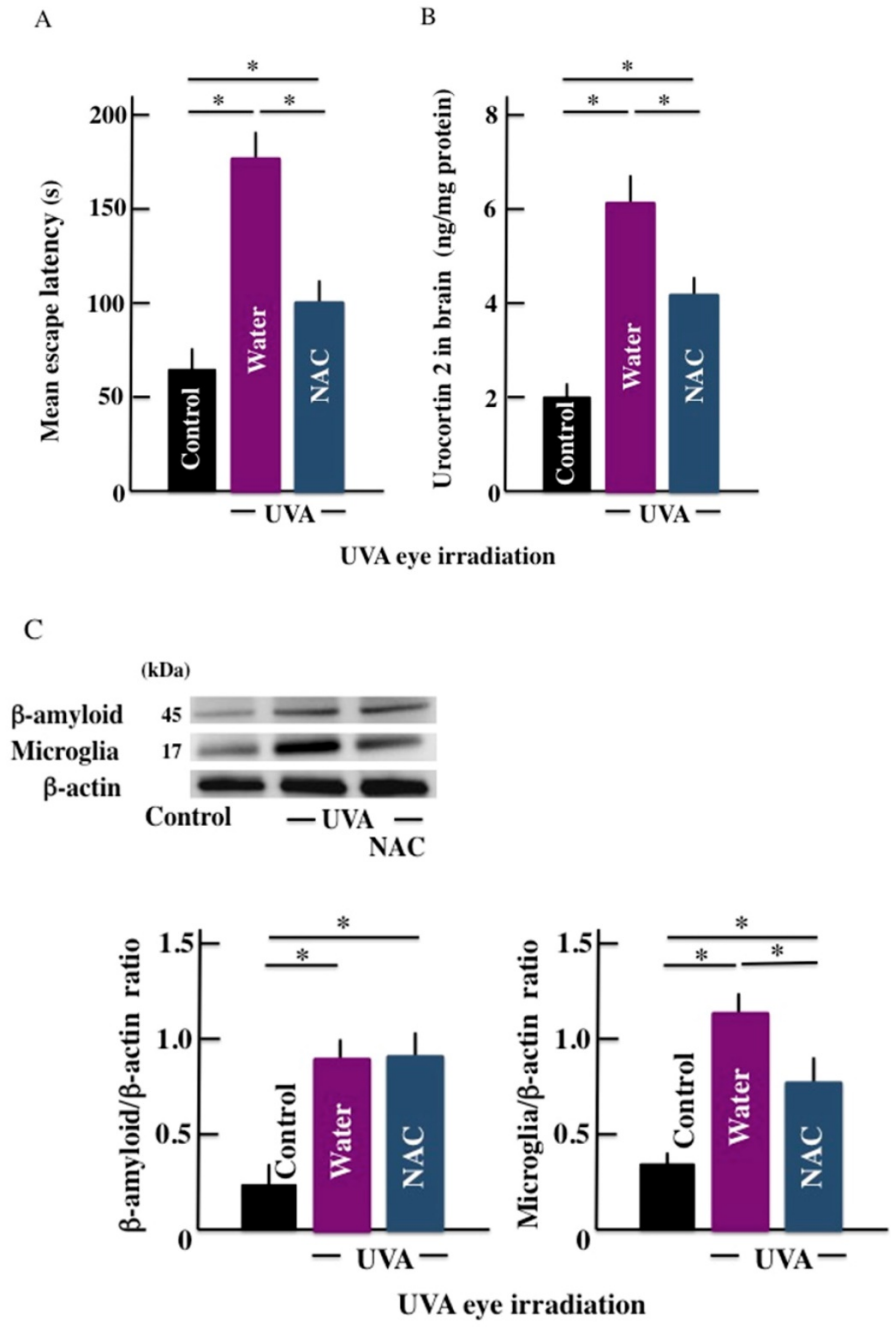
Effect of active oxygen scavenger (NAC) treatment on the long-term UVA eye irradiated mice. (A) memory and learning ability (mean escape latency), (B) urocortin 2 level in brain, and (C) the expression of β-amyloid and microglia in the brain. Values are expressed as the means ± SD derived from ten animals. *p < 0.05.

Long-term UVA eye irradiation significantly increased the mean escape latency (Fig. 7A). However, treatment with the hydroxyl radical scavenger inhibited the mean latency period. Similarly, the levels of β-amyloid, microglia marker, and urocortin 2 were higher in the brain of UVA eye irradiated mice the control mice. However, NAC injection in the UVA eye irradiated mice decreased the levels of urocortin 2 and microglia marker expression in the brain of the treated animals (Fig. 7B and 7C); however, the expression of β-amyloid remained unchanged (Fig. 7D).

## Discussion

The role of long-term UVA irradiation is well-known in causing chronic photooxidative stress and inflammation 18. CRH and urocortin 2 are the important mediators of the effects of physiological stress 19. CRH has a high affinity for CRHR-1 while urocortin binds to CRHR-2 with a high affinity 20. An increase in the levels of urocortin 2 levels have been reported in the brain of rats subjected to physiological stress, with a concomitant deterioration of memory and ability 21-23. In this study, we aimed to elucidate the effects of long-term UVA irradiation on urocortin 2 and CRHRs along with the memory and learning ability of mice.

Our results showed that the long-term UVA eye irradiation deteriorated memory and learning ability of the treated mice. This was associated with a parallel increase in the levels of ROS, CRH, CRHR-2 and urocortin 2 in the brain. We also observed that the effects were more pronounced in the animals subjected to eye irradiation than dorsal skin irradiation. The effects of the long-term UVA irradiation were ameliorated upon administration of CRHR-2 inhibitor as reflected by the results from the memory and learning ability examinations. On the other hand, the deterioration of memory and learning ability was observed when UVA non-irradiated animals were administered the CRHR-2 agonist. Furthermore, the impairment of memory and learning ability of long-term UVA eye irradiated mice was ameliorated by the administration of NAC, an active oxygen scavenger. Significantly, none of these interventions affected the expression of β-amyloid in the brain of animals subjected to the effects of long-term UVA radiation. Further, injecting CRHR-2 antagonist in animals subjected to long-term UVA exposure ameliorated the memory and learning ability impairment. Furthermore, our observation that injecting CRHR-1 agonist in control mice deteriorated their memory and learning ability validated our earlier findings. Collectively, these results indicate that urocortin 2 might be involved in long-term UVA eye exposure-mediated deterioration of the memory and learning ability. Although the antagonists and anti-ROS scavenging molecules used in this study could ameliorate the long-term UVA eye exposure-mediated impairment in memory and learning, it failed to return to the basal level under any of the treatment conditions.

In our study, the expression of the brain microglia markers increased remarkably by long-term UVA eye irradiation. Activated microglia releases inflammatory cytokine and chemokines, thereby inducing inflammation in the brain. In this study, we found that the brain of UVA eye irradiated mice showed a higher expression of macrophages markers and IL-6 than control (non-UVA) or UVA skin irradiated mice. The brain inflammation induces neurodegeneration and deteriorated memory and learning ability 24, 25. In our study, the expression level of the microglia remained unchanged in the animals treated with CRHR-1 agonist or inhibitor. This suggested that urocortin 2 may not be directly associated with the microglia expression. Interestingly, the expression level of the microglia marker decreased in the mice exposed to the long-term UVA eye irradiation and injected with the active oxygen scavenger. This raised the possibility that the UVA eye irradiation-induced active oxygen may promote the microglia expression, and reduce memory and learning ability. Further, injecting active oxygen scavenger inhibited the urocortin 2 levels in the brain of the treated animals. These results indicate that the long-term UVA eye irradiation-induced active oxygen may lead to an increase in the levels of urocortin 2. As the activated microglia further produces active oxygen 26, it may play an indirect role in increasing the expression levels of urocortin 2.

We also observed an increase in the levels of β-amyloid in the brain of animals exposed to long-term UVA eye irradiation. An accumulation of β-amyloid is known to causes Alzheimer's disease, and lead to the deterioration of memory and learning ability 27. In our study, long-term exposure to UVA eye irradiation caused an increase in the levels of β-amyloid, which could not be reversed by administration of active oxygen scavenger, CRHR-2 agonist, or antagonist. Our results were consistent with the earlier findings that that active oxygen enhances the expression of β-amyloid 28, 29. The chronic inflammation caused by long-term UVA eye irradiation can lead to the accumulation of β-amyloid. Our parallel results also showed that active oxygen could also upregulate the expression of microglia marker. Thus, our study correlates the increased microglia and β-amyloid expression with impaired memory and learning ability. However, we could not elucidate the association between the two, and further research is warranted to unravel the hidden explanations.

## Conclusion

To conclude, in this study, the deterioration of memory and learning ability was observed by the long-term UVA eye irradiation in mice. An increase in the levels of urocortin 2, microglia, and β-amyloid by the active oxygen or the chronic inflammation induced by UVA eye irradiation was observed. These results highlight the pathogenic role of UVA, considered innocuous so far, in causing neural deficits upon prolonged exposure. This also provides the proof-of-concept that a blanket shield from both, UVA and UVB is required, more so for the eyes, to prevent an increase in memory and learning related disorders in the future.
